# Family cumulative risk and meaning in life among Chinese secondary vocational school students: the mediating role of self-esteem and the moderating role of labor education support perception

**DOI:** 10.3389/fpsyg.2026.1882107

**Published:** 2026-09-08

**Authors:** Xuesong Du, Li Chen, Yanna Huang, Libo Jin, Lianjing Lu

**Affiliations:** 1 School of Education, South China Normal University, Guangzhou, China; 2 Shenzhen Institute of Education Sciences, Shenzhen, China; 3 The First Vocational Technical School of Shenzhen, Shenzhen, China; 4 Shenzhen Bao’an Vocational Technical School, Shenzhen, China; 5 Shenzhen Shajing Vocational High School, Shenzhen, China

**Keywords:** family cumulative risk, labor education support perception, meaning in life, secondary vocational school students, self-esteem

## Abstract

**Objective:**

This cross-sectional survey study investigated the association between family cumulative risk and meaning in life among Chinese secondary vocational school students, examining the mediating role of self-esteem and the moderating role of labor education support perception.

**Methods:**

A questionnaire survey comprising the Family Cumulative Risk Scale, Meaning in Life Questionnaire (MLQ), Rosenberg’s Self-Esteem Scale (RSES), and Labor Education Support Perception Scale (LESPS) was administered to 1536 secondary vocational school students from various grades.

**Results:**

Family cumulative risk was significantly and negatively associated with meaning in life (β = –0.34, *p* < 0.001), and the indirect association through self-esteem was significant. Labor education support perception, assessed using a newly developed scale with preliminary psychometric support, moderated the theoretically specified associations between family cumulative risk and self-esteem, and between self-esteem and meaning in life. Specifically, the negative association between family cumulative risk and self-esteem was stronger at higher levels of labor education support perception, consistent with the “drop in the bucket” model, although alternative explanations are also possible. The positive association between self-esteem and meaning in life was weaker at higher levels of labor education support perception, consistent with the “exclusion hypothesis.”

**Conclusion:**

Family cumulative risk was negatively associated with meaning in life, partially through lower self-esteem, and the strength of this indirect association varied as a function of labor education support perception. These findings identify a theoretically meaningful pattern of associations and highlight the importance of strengthening context-specific school-based support that helps secondary vocational school students develop self-esteem, construct positive vocational identities, and perceive their future work as meaningful.

## 1 Introduction

“Meaning in life” refers to an individual’s sense of the coherence of their existence, the exploration and commitment to life missions, and the pursuit and realization of valued goals (Baumeister, 1991; Steger et al., 2006). Meaning in life is an important protective factor for mental health, mitigating negative emotions such as depression and anxiety and enhancing well-being and life satisfaction (Baquero-Tomás et al., 2023; Halama and Dedova, 2007; Nell, 2014). China has increasingly emphasized cultivating meaning in life among adolescents in recent years. A joint action plan issued in 2023 by 17 government departments calls for the fostering of psychological qualities such as love of life, self-esteem, self-confidence, and optimism in students (Ministry of Education of the People’s Republic of China, 2023). This document affirms, at the national level, the importance of adolescent mental health, especially the cultivation of respect for life.

Among adolescent populations, Chinese secondary vocational school students constitute a particularly distinctive and under-studied group. China’s vocational education system is the largest of its kind globally, offering students pathways to pursue skills-based training rather than traditional academic routes. In 2025, 15.378 million students were enrolled in secondary vocational education, accounting for approximately half the number of general senior high school students (National Bureau of Statistics of China, 2026). Secondary vocational education in China is not merely an alternative form of schooling but an important component of the national high-school-stage education system, shaped by examination-based tracking after junior secondary school (Xue and Li, 2022). Many students enter secondary vocational schools after failing to obtain admission to general senior high schools, which may expose them to academic frustration, lowered social expectations, and stigmatized educational identities (Leng et al., 2025). Despite growing policy attention, the mental health status of this cohort remains concerning. Studies report prevalence rates of suicidal ideation ranging from 15.7 to 34.0% among vocational school students, significantly exceeding those among the general adolescent population (Gao et al., 2022; Wang et al., 2020). Compared with their peers in general high schools, secondary vocational school students are more likely to come from rural, migrant, or economically disadvantaged families, and they often face compounded pressures related to family resources, academic self-concept, future employment, and social recognition. Secondary vocational school students commonly experience a sense of meaninglessness and low self-esteem, and frequently lose their sense of self when pursuing life goals and values (Chen et al., 2023). These structural and developmental characteristics make them a special population for examining how family adversity is associated with positive psychological outcomes, such as meaning in life.

From a developmental perspective, secondary vocational school students are also situated in a critical period of vocational identity formation. Unlike students in general academic tracks, vocational students are expected to connect their current learning experiences with future occupational roles at a relatively early age. They must answer not only the general adolescent question of “Who am I?” but also the vocationally specific questions of “What kind of worker will I become?” and “How can others and society value my skills and labor?” Therefore, their meaning in life may be closely tied to whether they can construct a positive vocational identity and perceive their vocational pathway as valuable and socially meaningful. However, family cumulative risk may weaken this process by reducing emotional support, educational resources, and positive feedback, making it more difficult for students to develop a stable sense of self-worth and future purpose.

Moreover, the Chinese vocational education system, while massive in scale, has historically received less public investment and societal recognition than the general education track, further marginalizing its students. The present study therefore investigates the mechanisms associated with meaning in life among secondary vocational school students in Shenzhen city, China, providing theoretical and empirical support for interventions that enhance this sense of meaning in this population.

## 2 Literature review

### 2.1 Family cumulative risk and meaning in life

Ecological systems theory posits that the family is the most proximal and direct environmental system influencing adolescent psychological development (Bronfenbrenner and Morris, 1998). A 2024 report by the Ministry of Education’s Vocational Education Development Center indicated that more than 70% of secondary vocational school students come from rural or economically disadvantaged urban families, suggesting that children from families with higher cumulative risk are more likely to enter vocational schools (Ministry of Education’s Vocational Education Development Center., 2024). Family cumulative risk refers to the various factors within a family (e.g., low parental education levels and low family cohesion) that are detrimental to individual development and exert adverse effects through their cumulative and additive nature (Evans et al., 2013; Xiong et al., 2024). These risk factors operate from birth and produce sustained negative effects on individual development through long-term accumulation (Shonkoff et al., 2012). Existing research has linked higher numbers of adverse childhood experiences to lower meaning in life among vocational school students (Jing and Ding, 2024; Tang, 2023).

The family stress model extends these perspectives by specifying the psychological mechanisms through which family-level risks translate into children’s maladjustment. For example, parents with lower socioeconomic status are more prone to negative emotions such as anxiety and hostility, increasing parent–child conflict and negative parenting practices (Masarik and Conger, 2017). We therefore hypothesized that family cumulative risk was significantly and negatively associated with meaning in life among secondary vocational school students.

The cumulative risk model argues that an individual’s development is not influenced by a single risk factor, nor by the severity of risk or the duration of risk exposure, but rather by the number of risks they face (Evans et al., 2013). Constructing a comprehensive index of family risk can therefore more objectively reflect its association with vocational school students’ meaning in life. The present study constructed a family cumulative risk index across three dimensions–family structural risk, family resource deficiency risk, and family atmosphere risk–to further examine the association between family cumulative risk and students’ meaning in life. This study thus proposes Hypothesis 1: Family cumulative risk is negatively associated with meaning in life among secondary vocational school students.

### 2.2 The mediating role of self-esteem

The conservation of resources theory (Hobfoll, 2011) holds that individuals strive to obtain, maintain, and protect valued resources, and that the loss of or threat to resources triggers psychological crises. A supportive family environment, as an important external resource, can promote the accumulation and development of internal resources, such as self-esteem, which, in turn, positively influences meaning in life. Self-esteem refers to an individual’s internal evaluation of and attitude toward their self-worth, which is formed through feedback from others and social evaluation, reflecting their degree of self-acceptance and self-respect (Rosenberg, 2017). The lifespan perspective on self-esteem development argues that self-esteem is influenced by multiple systems, with the family environment being the most critical. A 27-year longitudinal study by Orth (2018) found that better family functioning facilitates the formation of a positive self-concept, of which self-esteem is a core component. Conversely, adverse family environments impair individuals’ self-representations, leading to low self-esteem (Orth et al., 2010).

Self-determination theory (Ryan and Deci, 2000) posits that individuals with high self-esteem feel satisfied with themselves, believe in themselves, possess positive self-cognition, and experience positive life events. Research has found significant correlations between self-esteem and meaning in life (Pérez-Fuentes et al., 2019). Parental care and support facilitate adolescents’ active outward exploration for meaning in life—individuals with high-quality parent–child attachment are more likely to establish stable connections with others and the external world, enhancing their sense of existence and thereby promoting the satisfaction of basic psychological needs through interaction with the environment, ultimately fostering meaning in life (Bodner et al., 2014; Zhao et al., 2023).

The majority of vocational students come from economically disadvantaged families that typically lack high-quality family educational resources and are unable to provide sufficient social support to meet their mental health needs. This makes them more susceptible to hopelessness and low self-esteem, thereby undermining their meaning in life. Secondary vocational school students may experience more frustration and criticism from the outside world than general high school students. If students perceive their vocational pathway as socially devalued, they may incorporate these negative evaluations into their self-concept, resulting in lower self-esteem. Conversely, when students view vocational learning as meaningful, skill-based, and socially contributive, they are more likely to develop a positive sense of self-worth. Thus, self-esteem can be understood as an internal psychological mechanism through which family resources and school experiences are translated into students’ evaluations of their vocational identities and, ultimately, their meaning in life. Hypothesis 2: Self-esteem mediates the relationship between family cumulative risk and meaning in life.

### 2.3 The moderating role of labor education support perception

School contexts can provide compensatory resources that buffer developmental risks for adolescents exposed to substantial family adversity (Benson et al., 2007). School-based resources may be particularly important for secondary vocational school students, as they often face not only family-related disadvantages but also external pressures associated with academic tracking and social evaluation. Among various school resources, labor education is especially relevant to vocational education. In China, labor education is not merely a practical skills curriculum but also a nationally mandated educational philosophy. According to official guidelines issued by the Ministry of Education of the People’s Republic of China (2020), labor education aims to cultivate students’ positive attitudes toward labor, work ethics, craftsmanship spirit, and appreciation of labor as a source of both personal and social value. For secondary vocational students, labor education is doubly significant: It serves as both a core component of their professional training and a potential psychosocial intervention that may compensate for family-based resource deficits (Qiu et al., 2023; Wu et al., 2024). Unlike general academic subjects, labor education provides hands-on, practice-oriented experiences that can foster a sense of competence, responsibility, and social contribution (Jiang, 2020). More importantly, labor education may function as an identity-relevant educational experience for vocational students. Through labor practice, skill training, productive activities, and service-oriented tasks, students can experience themselves as capable actors who are able to complete meaningful work, solve practical problems, and contribute to others. These experiences provide concrete feedback about competence and usefulness, which is particularly important for students who may have internalized academic frustration or social stigma associated with vocational schooling. According to identity-based motivation theory (Oyserman and Destin, 2010), when students perceive that their educational experiences are supportive of their future professional identities, they are more likely to invest in skill acquisition and develop a sense of purpose tied to their chosen field. This sense of purpose, in turn, enhances self-esteem by providing a positive self-definition: “I am someone who can contribute meaningfully through work.” By contrast, generic school support (e.g., academic tutoring or counseling) does not carry the same identity-relevant meaning for vocational students, making labor education support a more context-specific and theoretically compelling protective resource in this population. The present study, therefore, focuses on labor education support perception, defined as the support students perceive they receive from school-organized labor-related learning experiences, developmental guidance, and practice resources.

The emphasis on *perceived* support is theoretically important. Research has shown that perceived support may be more closely related to mental health outcomes than objective measures of support provided because it directly shapes individuals’ cognitive appraisal of available resources (Cohen and Wills, 1985). In the context of vocational education, students who perceive stronger labor education support may be more likely to interpret school experiences as meaningful, growth-promoting, and personally relevant. Labor education support perception is not merely a perception of school-provided resources; it also reflects students’ appraisal of whether the school offers opportunities to construct a positive vocational identity and recognize the personal and social value of their labor. Consequently, labor education support perception may be a more context-specific and ecologically valid protective factor for Chinese secondary vocational school students compared with general school support.

Student feedback is instrumental in assessing the effectiveness of labor education, as schools can use it to refine labor education activities, identify their strengths and areas for improvement, and ensure these programs are better aligned with students’ needs and interests (Xuede et al., 2023). The present study therefore developed a Labor Education Support Perception Scale to quantify students’ experiences and evaluations of school-based labor education, with the aim of examining its role in fostering students’ meaning in life and ultimately helping schools improve the overall quality of the educational experience.

The Risk and Protective Factors Model posits that protective factors mitigate risks by drawing on an individual’s internal psychological resources (Fergus and Zimmerman, 2005; Zimmerman et al., 2013). Within this framework, the compensatory model suggests that protective factors act independently and in opposition to risk factors; statistically, this manifests as a main effect of protective factors. The protective model proposes that protective factors moderate and attenuate the adverse effects of risk factors; statistically, this manifests as an interaction between protective factors and risk factors. There are two models within this protective framework: The first is the “helping in times of need” model, in which labor education support perception can buffer the negative effects of family cumulative risk; that is, the higher the perceived labor education support, the smaller the negative impact of family cumulative risk on self-esteem. The second is the “drop in the bucket” model, in which labor education support perception exerts a protective effect only when family cumulative risk is low; as risk levels increase, the protective effect of labor education support perception gradually weakens (Bao et al., 2014). These two interaction models carry distinct implications and offer different insights for practical interventions. The present study will examine whether vocational students’ perceptions of labor education support exert a risk-compensating or risk-protective effect on self-esteem. If a risk-protective effect exists, the study will determine whether it manifests as the helping in times of need model or the drop in the bucket model.

The “Protective-Protective Model” (Brook et al., 1999; Fergus and Zimmerman, 2005) posits that various protective factors may interact in two distinct ways when predicting developmental outcomes: the “facilitation hypothesis” and the “exclusion hypothesis” (Bao et al., 2013). If the facilitation hypothesis holds true, labor education support perception will more significantly promote meaning in life among individuals with high self-esteem than among those with low self-esteem. Conversely, if the exclusion hypothesis holds, labor education support perception will significantly enhance meaning in life for individuals with low self-esteem compared with those with high self-esteem. These different interaction modes also differ significantly in their practical implications. If the facilitation hypothesis is supported, then improving students’ labor education support perception becomes particularly important; only when labor education support perception is enhanced can students gain greater benefit from increased meaning in life. If the exclusion hypothesis is supported, mental health education should focus more on vocational school students with low self-esteem, working to continuously improve their labor education support perception and thereby enhance their meaning in life. Given these competing theoretical possibilities, rather than imposing a directional prediction, the present study treated the specific interaction pattern as an empirical question. Thus, this study proposes Hypothesis 3: Labor education support perception moderates the first-stage path (family cumulative risk → self-esteem) and the second-stage path (self-esteem → meaning in life) in the mediation model.

Despite growing evidence that family adversity is associated with poorer psychological adjustment in adolescents, relatively little is known about the impact of family cumulative risk on meaning in life, a positive existential outcome that has received less attention than psychopathology indicators. This gap may be particularly important for Chinese secondary vocational school students, who often face multiple developmental pressures arising from family disadvantage, academic tracking, and social evaluation. Although self-esteem is widely recognized as an important internal psychological resource, little is known about whether and under what conditions it explains the link between cumulative family adversity and meaning in life in this population. Finally, although school-based support is often considered a protective factor, few studies have examined whether a context-specific, practice-oriented form of school support—namely, labor education support perception—can shape the depletion of internal resources and their translation into meaning-related outcomes.

To address these gaps, the present study tested a theory-driven moderated mediation model linking family cumulative risk to meaning in life among secondary vocational school students in Shenzhen, China. Self-esteem was examined as a mediator, and labor education support perception was examined as a moderator of the first-stage path (family cumulative risk → self-esteem) and the second-stage path (self-esteem → meaning in life). Given the cross-sectional nature of the data, this model was intended to examine theoretically specified conditional associations rather than to establish causal pathways. This study thus makes three contributions to the literature. First, it extends the cumulative risk framework by shifting attention from predominantly negative mental health outcomes to meaning in life as a positive developmental and existential outcome among secondary vocational school students in Shenzhen. Second, it integrates conservation of resources and self-determination theories to clarify how family cumulative risk may be associated with lower meaning in life through lower self-esteem as an internal psychological resource. Third, it introduces labor education support perception, as operationalized by the newly developed LESPS, as a context-specific, school-based, practice-oriented resource in vocational education and examines its role across both theoretically specified stages of the mediation model, thereby offering a more nuanced account of when and for whom the indirect association is stronger or weaker.

The hypotheses, based on the theoretical reasoning reviewed above, are summarized as follows:

Hypothesis 1. Family cumulative risk is negatively associated with meaning in life among secondary vocational school students.

Hypothesis 2. Self-esteem mediates the relationship between family cumulative risk and meaning in life.

Hypothesis 3. Labor education support perception moderates the first-stage path (family cumulative risk → self-esteem) and the second-stage path (self-esteem → meaning in life) of the mediation model.

**FIGURE 1:**
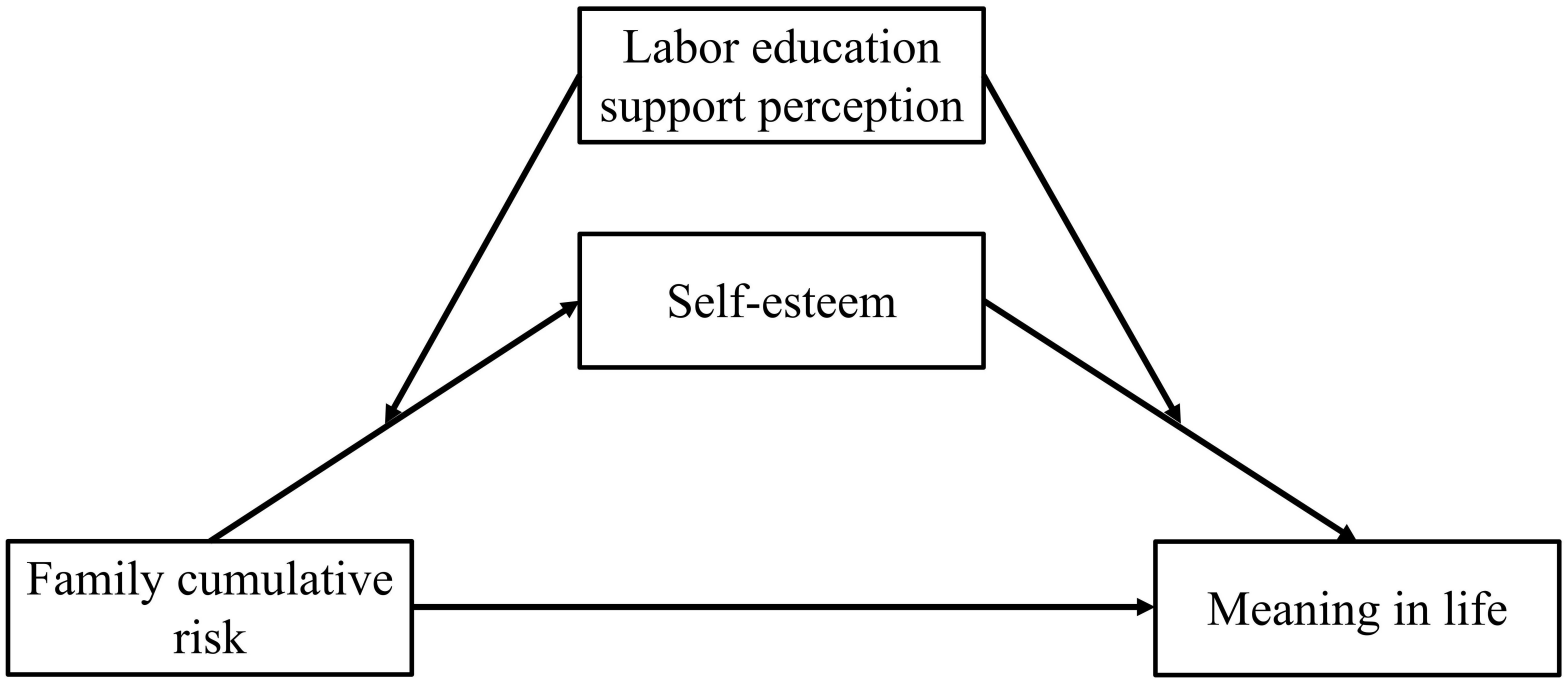
The proposed hypothesis model.

The hypothesized model is presented in Figure 1.

## 3 Materials and methods

### 3.1 Participants

A convenience sampling method was employed, involving 8 vocational schools (approximately 40% of all vocational schools in Shenzhen city) that participated in the survey. Shenzhen is among China’s most economically developed cities, with abundant educational resources and strong policy support for vocational and labor education. Therefore, the present sample represents secondary vocational school students in an economically developed urban context rather than all Chinese secondary vocational students. A total of 1947 questionnaires were collected. Following the removal of invalid questionnaires due to repetitive responses, failed attention-check tests, or excessively short response times, 1536 valid questionnaires remained, resulting in a valid response rate of 78.9%. Among them, 651 were males (42.4%) and 885 were females (57.6%), aged 15 to 19 years, with a mean age of 16.43 ± 0.84 years. The slightly higher proportion of females is consistent with the gender composition of the participating schools, where female students were overrepresented in several majors such as early childhood education and nursing. Although this ratio differs from the national secondary vocational school population, in which males slightly outnumber females, the present sample still covers a wide range of majors and grades. Class distribution was as follows: 750 students were in their first year of vocational school (48.8%), 534 students were in their second year (34.8%), and 252 students were in their third year (16.4%). Regarding residency, 349 students (22.7%) were Shenzhen residents, 757 students (49.3%) resided within Guangdong Province but outside Shenzhen city, and 430 students (28.0%) lived outside Guangdong Province.

With permission from classroom teachers and school counselors, data were collected during class breaks. The teacher displayed a QR code for the online survey (hosted on the Wenjuanxing platform) on the classroom screen, and students voluntarily completed the survey using their mobile phones. Participants were informed that this was a survey on the growth and development of vocational school students, that participation was anonymous and voluntary, that there were no right or wrong answers, and that they should answer truthfully based on their own circumstances. The average completion time was approximately 12 min. This study was approved by the Ethics Committee of Shenzhen Institute of Education Sciences. Written informed consent was obtained from all participants. For students under 18 years old, a parental/guardian informed consent form and an information sheet were distributed to the students at school and were taken home for their parents or legal guardians to read and sign. The signed forms were then returned to the school by the students prior to the survey. Only students who returned a signed parental consent form and who themselves provided written assent were included in the study. All procedures were conducted in accordance with the Declaration of Helsinki.

### 3.2 Measurement of variables

#### 3.2.1 Family cumulative risk measurement

Drawing on previous research (Bao et al., 2014; Buehler and Gerard, 2013; Xiong et al., 2020), a family cumulative risk index was constructed across three dimensions: family structure risk (family type, parent–child separation, and household registration status), family resource deficiency risk (parental educational attainment, parental employment, parental health, and family economic status), and family atmosphere risk (parental interaction and involvement, family cohesion, and parent–child relationship). Each risk indicator was dichotomized (0 = no risk, 1 = at risk) based on established cutoff criteria (see Table 1 for detailed criteria and risk proportions). The sum of all indicators constituted the individual’s family cumulative risk score, with higher scores indicating greater cumulative adversity. The specific coding rules for each indicator are provided in the Supplementary material.

**TABLE 1:** Criteria and proportions for assessing family cumulative risk among Chinese secondary vocational school students.

Risk type	*M* (*SD*)	Item	Criteria for assessing risk	Risk proportion
Family structure risk				
family type	–	1	“Biological father” and “biological mother” were not selected at the same time	15.10%
parent–child separation	–	2	One or both parents work away from home for extended periods	10.29%
household registration status	–	1	Non-local residents	77.28%
Family resource deficiency risk				
parental educational attainment	–	2	Both parents have an educational attainment level below “high school (including vocational high schools and technical schools)”	30.60%
parental employment	–	2	One or both parents do not have stable employment	22.27%
parental health	–	2	Either parent’s health status was “very poor” or “poor”	2.21%
family economic status	8.56 (2.18)	4	Score ≥ 75%	21.29%
Family atmosphere risk				
parental interaction and involvement	1.90 (1.01)	1	Score ≤ 25%	45.51%
family cohesion	56.00 (11.03)	16	Score ≤ 25%	26.37%
parent-child relationship	65.20 (7.51)	18	Score ≤ 25%	27.08%

#### 3.2.2 Meaning in life measurement

The study used the Meaning in Life Questionnaire (MLQ), developed by Steger et al. (2006), which comprises 10 items across two dimensions: the presence of meaning and the search for meaning. Items are rated on a 7-point Likert scale, ranging from 1 (absolutely untrue) to 7 (absolutely true). Higher scores indicate a higher level of meaning in life. The Cronbach’s α coefficients for the presence of meaning and search for meaning dimensions were 0.96 and 0.95, respectively, indicating high internal consistency reliability.

#### 3.2.3 Self-esteem measurement

The Rosenberg Self-Esteem Scale (RSES; Rosenberg, 1965) captures adolescents’ overall feelings regarding their self-worth and self-acceptance. It comprises 10 items rated on a 4-point Likert scale that ranges from 1 (*completely disagree*) to 4 (*completely agree*). Higher scores indicate higher self-esteem. Due to its simplicity and reliability, the RSES is widely employed to assess self-esteem across various age groups. The scale’s Cronbach’s α coefficient was 0.77.

#### 3.2.4 Labor education support perception measurement

Given the lack of a mature instrument specifically designed to assess secondary vocational school students’ perceptions of schools’ support for labor education, we developed the Labor Education Support Perception Scale (LESPS) for this study. The scale development process was guided by national and local policy documents on labor education, including the “*Guidelines for Labor Education in Primary, Secondary, and Higher Education Institutions (for Trial Implementation)*” (Ministry of Education of the People’s Republic of China, 2020) and the “*Implementation Guidelines of the Shenzhen Municipal Education Bureau on Further Strengthening Labor Education in Primary, Secondary, and Higher Education Institutions*” (Shenzhen Municipal Education Bureau, 2022). These documents emphasize labor values, labor literacy, practice opportunities, craftsmanship, responsibility, and integrating labor education with moral, intellectual, physical, and aesthetic development.

The initial item pool was generated through three sources: a review of policy documents and previous studies on labor education and vocational education, interviews with vocational school teachers who had experience organizing labor education activities, and discussions among members of the research team. The initial items were designed to capture students’ perceived support from school-organized labor-related learning experiences, developmental guidance, labor literacy cultivation, practice opportunities, and resource guarantees. After the initial item pool was created, items were reviewed for content relevance, clarity, developmental appropriateness, and suitability for secondary vocational school students by an expert panel (two professors in educational psychology and one senior vocational school administrator). Ambiguous, repetitive, or overly policy-oriented items were revised or removed.

To strengthen scale quality, we conducted a pilot survey at three schools prior to the formal survey. Based on item analysis, exploratory factor analysis, reliability analysis, and feedback from teachers and students in the pilot phase, the scale was revised and optimized. The final version of the LESPS comprises three dimensions. Dimension 1 is “Support for Labor-Based Education,” which includes 8 items and measures the extent to which schools implement the educational goal of “integrating the five aspects of education” (moral, intellectual, physical, aesthetic, and labor education) in their labor education programs. This refers to the extent to which students perceive the school’s efforts to promote their all-round development through labor education. Specifically, it assesses whether the school uses labor education to cultivate students’ sense of responsibility and teamwork (moral education), enhance their hands-on skills and problem-solving abilities (intellectual development), strengthen their physical fitness and willpower (physical education), and foster aesthetic appreciation and an appreciation for the fruits of labor (aesthetic education^1^).

Dimension 2 is “Support for Cultivating Labor Literacy,” comprising 11 items that measure the effectiveness of schools’ practices in cultivating the core elements of labor literacy. This refers to students’ perceptions of the school’s efforts to cultivate their labor literacy, specifically including whether the school helps them form correct attitudes toward labor (respecting labor, loving labor, and recognizing that labor creates a better life), whether it fosters a positive work ethic (thrift, diligence, innovation, and dedication), and whether it promotes the spirit of model workers (professional identity and dedication to one’s job) and the spirit of craftsmanship (pursuit of perfection and excellence). In line with the distinctive features of secondary vocational education, this dimension emphasizes measuring the spirit of model workers and craftsmanship to reflect vocational education’s emphasis on professional identity and the pursuit of technical excellence.

Dimension 3 is “Support for Labor Practice and Resources,” comprising 12 items and measuring the extent of support provided by the school in terms of conditions for implementing labor education and resource allocation. This refers to the students’ perceptions of the support provided by the school in terms of labor practice opportunities and resource guarantees, specifically including whether the school offers practical opportunities for the three types of labor (daily life labor, productive labor, and service-oriented labor), whether labor education is organized and implemented through multiple channels (labor courses, subject integration, practical activities, and home-school collaboration), and whether adequate support conditions are provided (labor tools and venues, safety education, and evaluation feedback).

Items were rated on a 5-point Likert scale, ranging from 1 (completely inconsistent) to 5 (completely consistent). The higher the score, the greater the student’s perception of the school’s labor education support. In the formal study, confirmatory factor analysis results indicated an acceptable model fit: χ^2^/*df* = 3.52, CFI = 0.91, TLI = 0.90, RMSEA = 0.08, SRMR = 0.04. To provide preliminary evidence of discriminant validity, we examined correlations between the LESPS total score and scores on the other three scales used in this study. The correlations ranged from 0.34 to 0.57, all below the 0.85 threshold, suggesting that the LESPS was not redundant with the other constructs measured in this study (Kline, 2015). The Cronbach’s α coefficient for the LESPS was 0.98, and the Cronbach’s α coefficients for the three dimensions were 0.96, 0.96, and 0.95, respectively. Nevertheless, because the LESPS is a newly developed scale and was validated only within the present sample, the psychometric evidence should be regarded as preliminary. Further validation in independent samples is required before firm conclusions can be drawn about the construct validity, stability, and generalizability of labor education support perception as measured by this scale.

### 3.3 Data analyses

Data were analyzed using SPSS 26.0, employing common method bias tests, reliability and validity tests, descriptive statistics, and correlation analyses. The mediating and moderating roles of self-esteem and labor education support perception were examined using Models 4 and 58 in the SPSS macro PROCESS 3.3 plugin (Hayes, 2017).

## 4 Results

### 4.1 Common method bias

During the data analysis phase, Harman’s single-factor test was used as a preliminary diagnostic check for common method variance (Podsakoff et al., 2003). The results showed that six factors had eigenvalues greater than 1, and the variance explained by the largest common factor was 28.96%, below the commonly used threshold of 40% (Zhou and Long, 2004). This result suggests that a single common factor did not dominate the variance in the measured items. However, because Harman’s single-factor test is limited in its ability to detect common method bias, and all variables were measured using self-report questionnaires at a single time point, common method variance cannot be fully ruled out.

### 4.2 Descriptive statistics and correlation analysis

The results of descriptive statistics and correlation analysis are shown in Table 2. The mean score of Shenzhen vocational school students’ meaning in life was 5.25 (out of 7), which is above average. Family cumulative risk was significantly negatively correlated with self-esteem, labor education support perception, and meaning in life. Self-esteem, labor education support perception, and meaning in life were all significantly positively correlated.

**TABLE 2:** Descriptive statistics and correlations among study variables (*N* = 1536).

Variable	*M*	*SD*	1	2	3	4
1. Family cumulative risk	2.09	1.82	1	1	1	1
2. Self-esteem	2.89	0.49	–0.33***			
3. Meaning in life	5.25	1.03	–0.34***	0.52***		
4. Labor education support perception	4.04	0.62	–0.34***	0.44***	0.57***	

***p < 0.001.

In addition, we examined differences in core variables across demographic variables. The independent-samples *t*-test revealed significant gender differences in meaning in life (*t* = 4.12, *df* = 1534, *p* < 0.001, Cohen’s *d* = 0.21), with males reporting significantly higher levels than females. The one-way ANOVA results show that self-esteem, labor education support perception, and meaning in life varied significantly across different grades [*F*_(2,1533)_ = 3.67, *p* = 0.026, η^2^ = 0.005; *F*_(2,1533)_ = 8.62, *p* < 0.001, η^2^ = 0.011; *F*_(2,1533)_ = 3.06, *p* = 0.047, η^2^ = 0.004]. Therefore, gender and grade were controlled for in subsequent analyses.

### 4.3 Testing the moderated mediation model

To reduce multicollinearity, all predictor variables were standardized in the regression analysis (Fang et al., 2015). Model 4 in the PROCESS package was used to test for mediation effects, with gender and grade serving as control variables. Family cumulative risk was directly and negatively associated with meaning in life (β = –0.34, *t* = –14.15, *p* < 0.001), and significantly and negatively associated with self-esteem (β = –0.33, *t* = –13.73, *p* < 0.001). Family cumulative risk and self-esteem were both significantly associated with meaning in life (β = –0.19, *t* = –8.32, *p* < 0.001; β = 0.46, *t* = 20.24, *p* < 0.001). The mediating effect of self-esteem accounted for 44.67% of the total effect, with a 95% confidence interval of [-0.18, -0.13]. Self-esteem therefore partially mediates the relationship between family cumulative risk and vocational school students’ meaning in life in the present Shenzhen sample.

**FIGURE 2:**
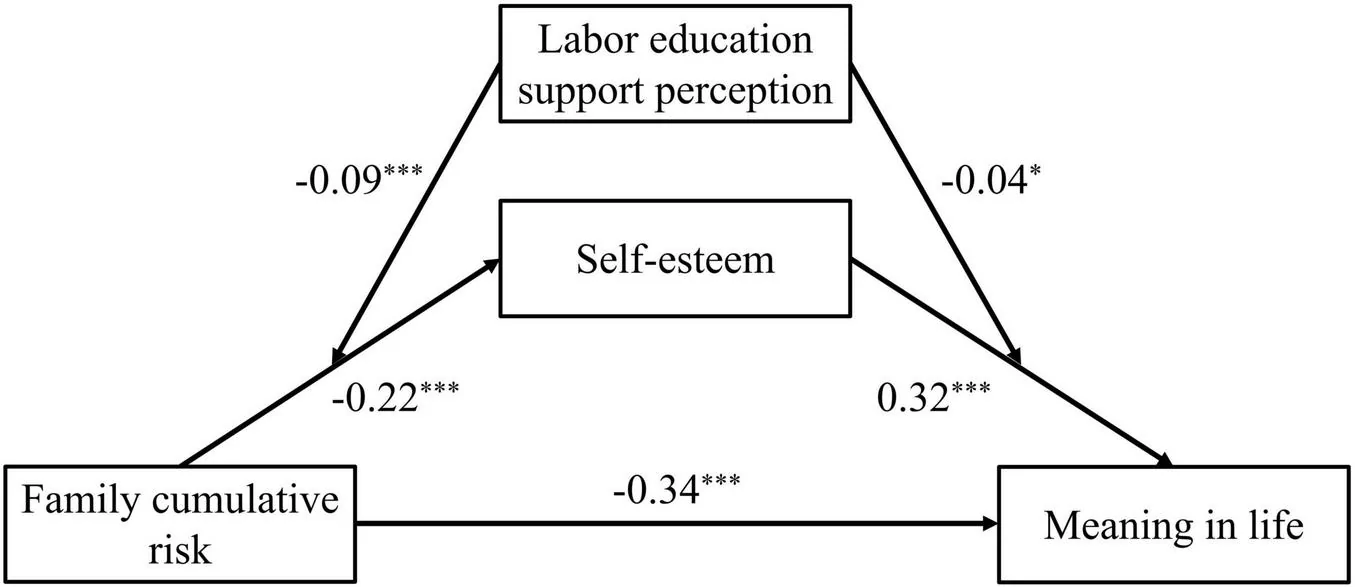
The moderated mediation model of the relationship between family cumulative risk and meaning in life. *p < 0.05, ***p < 0.001.

Next, Model 58 was used to further examine the moderating effect of labor education support perception, with family cumulative risk as the independent variable, meaning in life as the dependent variable, self-esteem as the mediating variable, labor education support perception as the moderating variable, and gender and grade as the control variables. The results are presented in Figure 2 and Table 3. The interaction term between family cumulative risk and labor education support perception was significantly and negatively associated with self-esteem (β = –0.09, *t* = –4.08, *p* < 0.001); the interaction term between self-esteem and labor education support perception was also significantly and negatively associated with meaning in life (β = –0.04, *t* = –2.42, *p =* 0.016). Therefore, labor education support perception moderates the first half (family cumulative risk → self-esteem) and the second half (self-esteem → meaning in life) of the mediating path.

**TABLE 3:** Results of the moderated mediation analysis (*N* = 1536).

Outcome variable	Predictor variable	β	*SE*	*t*	95% CI	*R* ^2^	*F*
Self-esteem	Gender	0.01	0.05	0.02	[–0.09, 0.09]	0.24	96.13***
	Grade	0.08	0.03	2.67**	[0.02, 0.14]		
	Family cumulative risk	–0.22	0.02	–9.16***	[–0.27, –0.17]		
	Labor education support perception	0.38	0.02	15.81***	[0.33, 0.43]		
	Family cumulative risk × labor education support perception	-0.09	0.02	–4.08***	[–0.14, –0.05]		
Meaning in life	Gender	–0.18	0.04	–4.53***	[–0.25, –0.10]	0.43	193.76***
	Grade	0.04	0.03	1.51	[–0.01, 0.09]		
	Family cumulative risk	–0.10	0.02	–4.50***	[–0.14, –0.05]		
	Self-esteem	0.32	0.02	14.34***	[0.28, 0.37]		
	Labor education support perception	0.40	0.02	17.85***	[0.35, 0.44]		
	Self-esteem × labor education support perception	–0.04	0.02	–2.42*	[–0.07, –0.01]		

*p < 0.05, **p < 0.01, *p < 0.001.

**FIGURE 3:**
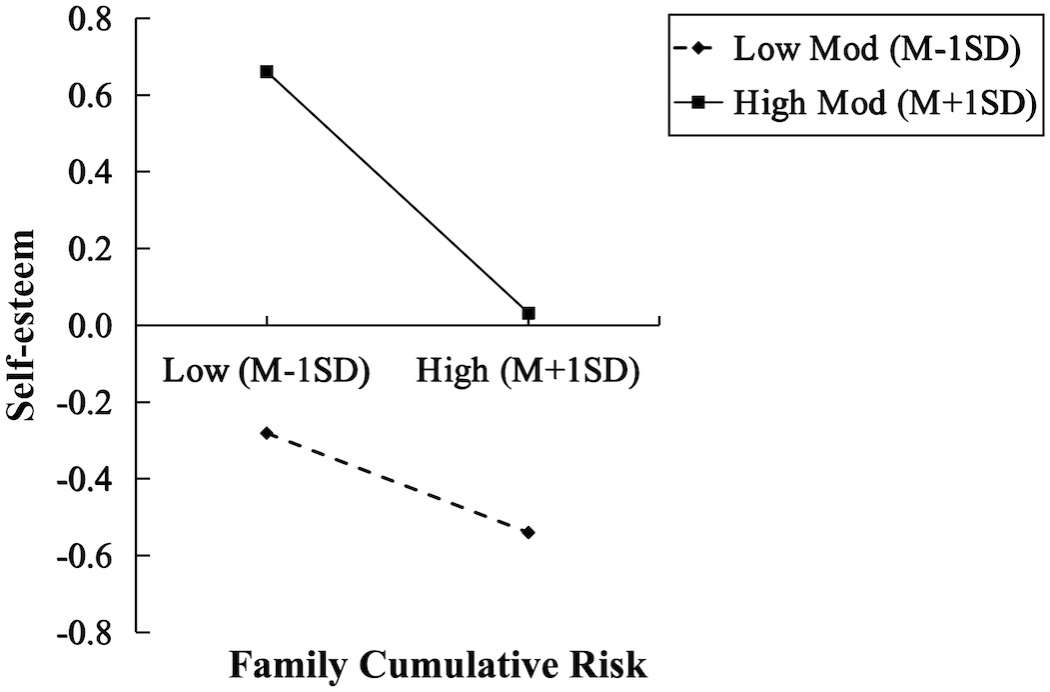
The moderating effect of labor education support perception between family cumulative risk and self-esteem.

We conducted a simple slope analysis of the moderating effect of labor education support perception (Hayes and Montoya, 2016), dividing participants into high and low groups based on scores that were one standard deviation above or below the mean. Regarding the moderating effect on the first half of the path (see Figure 3), the results showed that when labor education support perception scores were low (*M*-1*SD*), the negative association between family cumulative risk and self-esteem was significant (β = –0.13, *t* = –4.32, 95% CI = [–0.19, –0.07]), and when labor education support perception scores were high (*M*+1*SD*), this negative association was stronger (β = –0.31, *t* = –8.68, 95% CI = [–0.39, –0.24]). This interaction pattern indicates that the negative association between family cumulative risk and self-esteem was stronger among students who reported higher labor education support perception. This pattern is counterintuitive and should be interpreted cautiously. One possible interpretation is the drop in the bucket model: perceived labor education support may be beneficial under relatively low levels of family cumulative risk, but may be insufficient to offset the disadvantages associated with higher levels of cumulative family adversity. However, this interpretation is not the only plausible explanation. Therefore, this finding should not be interpreted as indicating that greater labor education support perception is harmful; rather, one possible interpretation is that the protective value of labor education support may become insufficient as family risk accumulates.

**FIGURE 4:**
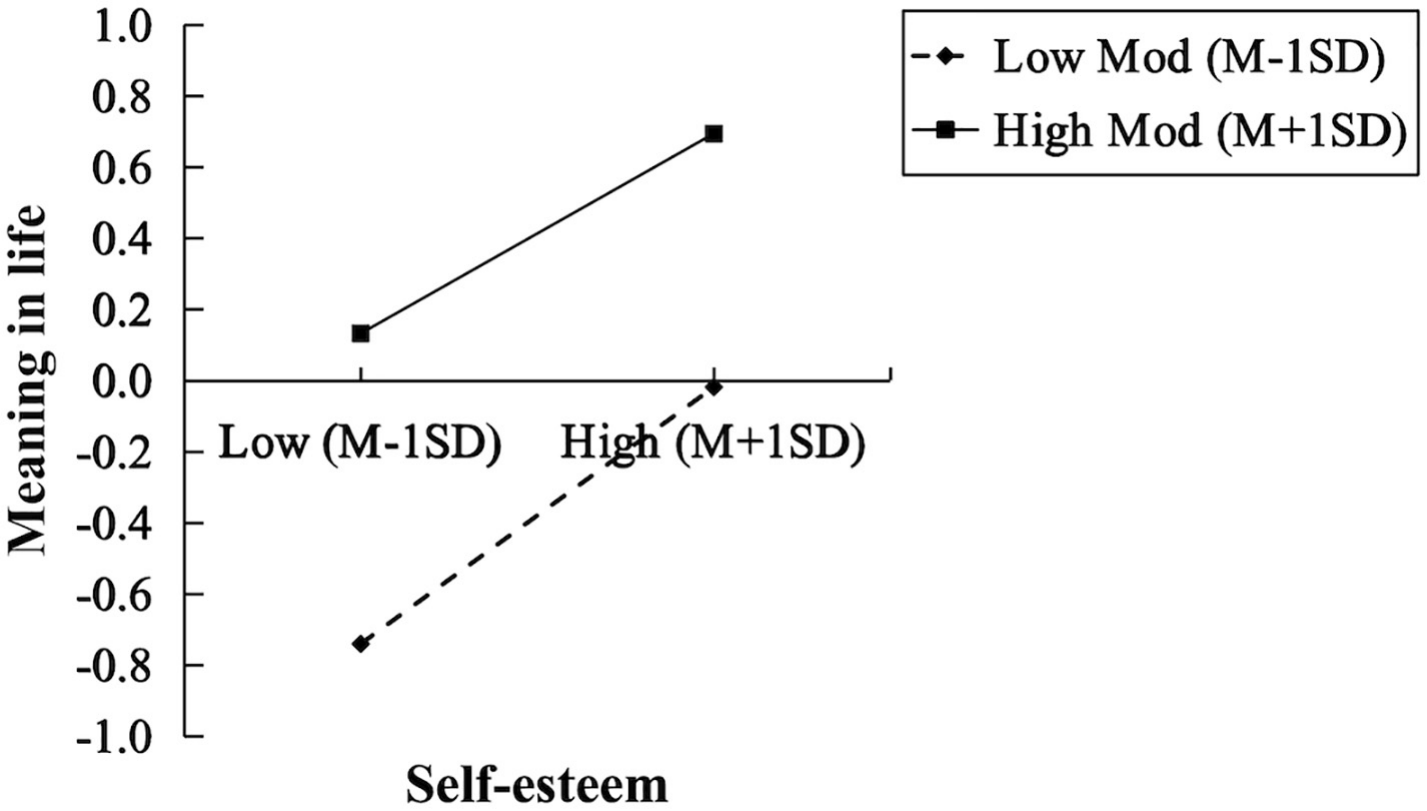
The moderating effect of labor education support perception between self-esteem and meaning in life.

Regarding the moderating effect on the second part of the path (see Figure 4), the results indicated that when labor education support perception was low (*M*-1*SD*), the positive association between self-esteem and meaning in life was significant (β = 0.36, *t* = 11.94, 95% CI = [0.30, 0.42]), whereas when labor education support perception was high (*M*+1*SD*), this positive association was weaker (β = 0.28, *t* = 11.14, 95% CI = [0.23, 0.33]). The association between self-esteem and meaning in life varied in intensity depending on the level of perceived labor education support. The positive association between labor education support perception and meaning in life was stronger in individuals with low self-esteem. The mediating effect of self-esteem on the relationship between family cumulative risk and meaning in life at different levels of labor education support perception is shown in Table 4. The moderated mediating effects were significant at different perceived levels of labor education support, at -0.05, -0.07, and -0.09, respectively.

**TABLE 4:** Mediation effect values at different levels of labor education support perception.

Labor education support perception	Effect	Boot *SE*	95% CI
*M*–1*SD*	–0.05	0.01	[–0.07, –0.02]
*M*	–0.07	0.01	[–0.09, –0.05]
*M*+1*SD*	–0.09	0.02	[–0.12, –0.06]

## 5 Discussion

### 5.1 The relationship between family cumulative risk and meaning in life

Consistent with previous research (Chen et al., 2023; Ma et al., 2022), students in the present Shenzhen vocational school sample reported a slightly above-average meaning in life, indicating that this group is in a critical stage of actively exploring the value of life. Steger et al. (2006) suggested that meaning includes “presence of meaning” and “search for meaning.” Vocational school students are in a critical period of identity development and inherently exhibit a strong inclination to explore their identity and future direction, leading to high levels of engagement in the “search” dimension of life meaning. Furthermore, in Chinese culture, collectivist values—rooted in Confucian traditions and reinforced through family socialization and school moral education—emphasize the individual’s role in maintaining social order and family relationships. These values provide adolescents with relatively clear reference points for understanding their place in the family and society, thereby helping to establish a stable foundation for a sense of meaning (Lambert et al., 2013).

Regarding demographic variables, our findings showed that males reported significantly higher levels of meaning in life than females, consistent with previous findings (Kaya et al., 2023), possibly stemming from differences in gender role expectations and socialization. Additionally, students in higher grades reported higher levels of meaning in life, possibly related to their greater experience in career exploration and practice (Xiang et al., 2024). Furthermore, consistent with the findings of Yu et al. (2025), our study did not find a significant difference in meaning in life by “household registration” (hukou, the official household registration system in China that classifies individuals as agricultural or non-agricultural and affects access to public services and educational resources). Although the family serves as the initial microsystem for secondary vocational school students, the school environment gradually becomes an equally important microsystem upon enrollment. In the Shenzhen context, secondary vocational schools tend to uniformly allocate educational resources, including practical training, extracurricular activities, and career guidance, to all students. Consequently, a similar educational environment constitutes an external mechanism that “neutralizes” differences in household registration status, allowing students from diverse backgrounds to access the same quality of education and opportunities for personal and professional development.

Our results indicate that family cumulative risk was negatively associated with vocational school students’ meaning in life in the present Shenzhen sample, supporting Hypothesis 1. The family functioning model (Miller et al., 2000) suggests that healthy family functioning promotes children’s positive psychosocial adaptation and profoundly shapes the development of their future behavioral patterns. Conversely, dysfunctional family behaviors (such as parental alienation) may reduce students’ proactive attitude toward exploring their surroundings, thereby affecting their mental health development. In this Shenzhen sample, secondary vocational school students who report more adverse family factors and poorer family functioning tend to report lower levels of meaning in life. This pattern is consistent with the family stress model, although causal direction cannot be confirmed in the present cross-sectional design. In families with more risk factors, parents experience greater stress, leading to a series of negative emotions and passive parenting styles (Gard et al., 2020). This process may be associated with feelings of neglect or harm among vocational school students and with lower meaning in life.

### 5.2 The mediating role of self-esteem

Our findings supported Hypothesis 2: Self-esteem partially mediated the relationship between family cumulative risk and meaning in life, with the indirect effect accounting for approximately 44.67% of the total effect in the present Shenzhen sample. This is consistent with the theoretical framework proposed in the introduction. From the perspective of conservation of resources theory (Hobfoll, 2011), family cumulative risk may represent a chronic depletion of external resources that is associated with lower internal psychological resources, such as self-esteem. From the perspective of self-determination theory (Ryan and Deci, 2000), diminished self-esteem compromises the satisfaction of basic psychological needs—particularly competence and relatedness—thereby reducing individuals’ capacity to experience and construct meaning in life.

Self-esteem is a core component of the self system and plays a protective role in individual development (Moksnes and Reidunsdatter, 2019). Consistent with previous research, we found that self-esteem levels were significantly linked to an individual’s sense of meaning in life; individuals with higher self-esteem tend to have greater well-being and stronger meaning in life (Gori et al., 2022; Kaya et al., 2023; Yun et al., 2025). As an internal developmental resource, self-esteem mediates the relationship between family cumulative risk and meaning in life among students in the Shenzhen sample. On the one hand, vocational school students experience insecurity after exposure to negative family factors, which are integrated into their self-construction and affect their self-esteem levels. On the other hand, individuals with lower self-esteem may be more likely to process negative information in a self-relevant manner, which may be associated with lower meaning in life.

### 5.3 The moderating role of labor education support perception

Our results showed that labor education support perception moderated the first and second halves of the mediation model, supporting Hypothesis 3. More specifically, labor education support perception moderated the association between family cumulative risk and self-esteem, as well as the association between self-esteem and meaning in life. These findings suggest that, rather than operating uniformly across developmental contexts, the impact of school-based, practice-oriented support depends on the level of family risk and students’ internal psychological resources.

In the first half of the model, labor education support perception served risk-compensation and risk-protection functions. The negative association between family cumulative risk and self-esteem was stronger among students reporting higher labor education support perception, compatible with the drop in the bucket model. That is, under low family cumulative risk, stronger perceived labor education support is associated with higher students’ self-esteem; however, when family cumulative risk is high, the damage to students’ core psychological resources (such as self-esteem) reaches a “ceiling effect,” making it difficult for even strong external support from schools to fully compensate for it (Cohen and Wills, 1985). In other words, labor education support perception is not “the more, the worse,” but rather “insufficient” in the face of high risk. In this sense, labor education support perception appears to function as a meaningful but limited protective resource, the buffering effect of which weakens as family risk intensifies. When family adversity accumulates across structural, resource-related, and relational domains, school-based labor education alone may be too limited to compensate for the chronic depletion of family resources.

Several alternative explanations should also be considered. First, students exposed to substantial family adversity may require more intensive and multi-system interventions than labor education alone can provide. Family cumulative risk in the present study included not only structural disadvantages but also family atmosphere risks, such as limited parental involvement and poorer parent–child relationships. These risks may directly threaten adolescents’ sense of worth, belonging, and emotional security (Buehler and Gerard, 2013; Fritz et al., 2018). Although labor education can provide competence-based and practice-oriented support, it may not fully address the emotional injuries and relational insecurity associated with severe family adversity. Therefore, labor education support needs to be integrated with counseling services, teacher mentoring, family-school collaboration, and community support to benefit high-risk students. Second, a contrast effect may be operating. Social-comparison research indicates that individuals differ in their tendency to compare themselves with relevant standards and others (Gibbons and Buunk, 1999). Students who perceive strong labor education support at school may become more aware of the discrepancy between supportive school experiences and insufficient family resources. For students with high family cumulative risk, supportive school contexts may make family deficits more salient by contrast, thereby strengthening the association between family adversity and lower self-esteem. Taken together, the present finding should not be interpreted as suggesting that higher labor education support perception is detrimental. Rather, it indicates that labor education support perception may have conditional and bounded protective value. It may support self-esteem among students with relatively low family cumulative risk, but may be insufficient, mismatched, or experienced differently among students exposed to multiple family adversities. Future longitudinal and intervention studies are needed to distinguish among these competing explanations.

In the latter half of the pathway, the association between self-esteem and meaning in life varied in intensity depending on the level of labor education support perceived. Individual–environment interaction theory posits that developmental outcomes are determined by the interaction between individual traits and the environment (Lerner, 2004). Labor practice may temper the will of vocational school students and cultivate their self-esteem, independence, and self-reliance. Therefore, self-esteem and support perception, as individual and environmental factors, may jointly influence the meaning in life experienced by vocational school students (Wang et al., 2024). The positive association between labor education support perception and meaning in life was stronger in individuals with low self-esteem in our sample, consistent with previous research (Xu and Niu, 2024). This interaction mode does not support the facilitation hypothesis (i.e., that the enhancement of meaning in life by labor education support perception is more pronounced in individuals with high self-esteem), but rather the exclusion hypothesis, that is, when an individual’s internal resources (self-esteem) are sufficient, the role of external support may be relatively weaker; when internal resources are scarce, the compensatory effect of external support is more prominent. Specifically, for individuals with high self-esteem, the marginal utility of additional external support (labor education) on their meaning in life is low, whereas for those with low self-esteem, the external resource of perceived labor education support may compensate for their lack of internal resources, significantly enhancing their meaning in life. From the perspective of vocational identity development, this pattern may indicate that students with low self-esteem have not yet established a stable and positive vocational identity. For these students, supportive labor education experiences may provide concrete evidence that they are capable, useful, and able to contribute through labor and skills. Such experiences may compensate for their fragile self-evaluation and help them connect present learning with future occupational goals, thereby enhancing meaning in life. In contrast, students with high self-esteem may already possess relatively stable internal resources and a clearer sense of personal worth, so additional perceived labor education support produces a smaller incremental effect.

The moderated mediation model in the present study thus paints a nuanced picture of how family cumulative risk is associated with meaning in life among secondary vocational school students in Shenzhen. The direct association suggests that accumulated family adversity is linked to lower meaning in life, beyond the indirect association through self-esteem. The indirect association through self-esteem highlights the role of internal psychological resources in translating environmental risk into existential outcomes. Importantly, the two distinct moderation patterns—drop in the bucket for the first stage and exclusion hypothesis for the second stage—carry different implications for intervention. For vocational students facing high family cumulative risk in Shenzhen, school-based labor education support alone may be insufficient to buffer the damage to self-esteem, suggesting a need for family-level and community-level interventions. For students with low self-esteem, however, labor education support perception may serve as a compensatory resource that is more strongly associated with meaning in life, suggesting that targeted labor education programs should prioritize this subgroup.

It is also important to interpret these findings in light of the preliminary status of the LESPS. Although the scale showed high internal consistency and acceptable factorial validity in the present sample, it remains a newly developed instrument. Therefore, the observed moderating patterns may partly depend on how labor education support perception was operationalized in this study. The construct may overlap with broader perceptions of school support, vocational identity support, or access to practical training resources. Further psychometric work is needed to clarify the boundaries of this construct and to determine whether the same factor structure and associations can be replicated in independent samples.

### 5.4 Alternative theoretical explanations

Although the hypothesized model was grounded in ecological systems theory, the family stress model, conservation of resources theory, and self-determination theory, the cross-sectional design does not allow us to establish temporal or causal ordering. Therefore, the findings should be interpreted as evidence of theoretically meaningful associations rather than causal pathways. Several alternative explanations may be considered.

First, the reverse association is plausible. Cognitive models propose that enduring beliefs about the self and others can influence the selection and interpretation of interpersonal information (Beck and Haigh, 2014). Students with lower self-esteem or lower meaning in life may evaluate their family environment more negatively. Students with low self-esteem may be more sensitive to rejection, conflict, or lack of support and may therefore perceive parent–child relationships, family cohesion, or parental involvement more negatively. Similarly, students with lower meaning in life may have difficulty constructing a coherent and purposeful life narrative, which may lead them to interpret family experiences as less supportive or more stressful.

Second, the reciprocal developmental process is also possible. Longitudinal evidence indicates that adolescent psychological adjustment and perceived parent–child relationship quality can influence each other over time. In a four-wave study of 1313 adolescents, depressive symptoms predicted later lower perceived relationship quality with parents, while perceived relationship quality with mothers also predicted later depressive symptoms (Branje et al., 2010). Likewise, five-wave data from 9770 children showed bidirectional associations between parental rejection/warmth and peer victimization, with child maladjustment implicated in these cross-domain processes (Kaufman et al., 2019). These studies do not test the exact constructs examined here, but they demonstrate that young people’s psychological functioning and their relational environments may be dynamically intertwined. Thus, family cumulative risk, self-esteem, and meaning in life may be reciprocally related across adolescence. For example, family adversity may undermine self-esteem and meaning in life, whereas lower self-esteem or meaning in life may be accompanied by emotional withdrawal, reduced communication, or more negative interpretations of family interactions, which could subsequently erode relationship quality or its perception.

Third, labor education support perception may also be subject to appraisal bias. Relational regulation theory emphasizes that perceived support is embedded in relational and cognitive processes rather than being equivalent to the amount of support objectively available (Lakey and Orehek, 2011). Students with higher self-esteem or stronger meaning in life may be more likely to recognize, internalize, or report labor education experiences as supportive, whereas students with fewer psychological resources may appraise the same experiences differently. Therefore, the observed interactions should be understood as conditional associations among family risk, self-esteem, meaning in life, and labor education support perception, rather than as definitive evidence that labor education support perception causally changes the strength of developmental pathways.

Although alternative directional explanations are possible, the present study positioned family cumulative risk as the theoretically specified antecedent for three reasons. First, several components of family cumulative risk, such as family type, parental education, parental employment, parental health, and family economic status, are relatively stable contextual conditions that are unlikely to be caused by students’ current meaning in life. Second, ecological systems theory and the family stress model suggest that family conditions constitute proximal developmental contexts that shape adolescents’ self-evaluation and psychological adjustment. Third, previous longitudinal studies have suggested that supportive family functioning predicts adolescents’ later self-esteem and psychological well-being (Krauss et al., 2020; Shek, 2005), while family socioeconomic resources are prospectively associated with adolescents’ mental health, self-esteem, and broader psychosocial adjustment (Hosokawa and Katsura, 2017; Zeng et al., 2026). Nevertheless, because some family atmosphere indicators were self-reported, students’ psychological states may have influenced their perceptions of family risk. Therefore, the present findings should be interpreted as consistent with, but not conclusive evidence for, the proposed directional model.

## 6 Implications

From a practical perspective, the present findings are most directly relevant to secondary vocational schools in Shenzhen and other relatively resource-rich urban contexts. In such contexts, interventions may focus not only on increasing the amount of labor education schools provide but also on strengthening students’ perceptions that such experiences are supportive, meaningful, and developmentally relevant. Based on the three dimensions proposed in the preliminary LESPS, schools in similar resource-rich urban contexts may consider implementing the following strategies. First, in terms of development-oriented support, labor education activities may explicitly incorporate psychoeducational goals, such as responsibility, teamwork, persistence, and problem solving, and teachers may make these goals visible through guidance and structured reflection. Second, in terms of labor literacy support, schools may connect labor experiences with values, such as occupational dignity, craftsmanship, and contribution, so that students can interpret labor as a source of identity development and personal worth rather than merely task completion. Third, in terms of practice and resource support, schools in contexts with sufficient labor education infrastructure should strive to provide students with access to diverse, safe, and well-supported practice opportunities, alongside timely feedback, recognition, and opportunities for success.

Importantly, within the Shenzhen context examined in this study, the findings suggest that labor education support may need to be differentiated across student groups. For students facing high family cumulative risk, labor education support should not be treated as a stand-alone intervention. Practice-oriented school support may be insufficient when students experience accumulated family disadvantages, particularly those involving low family cohesion, poor parent–child relationships, economic hardship, or parental absence. Therefore, labor education may be more beneficial when embedded within a broader support system that includes psychological counseling, teacher mentoring, family-school communication, peer support, and, when necessary, community-based social services. Such integrated interventions may better address both competence-related needs and relational-emotional needs among high-risk vocational students in similar urban vocational school contexts. For students with low self-esteem, however, labor education may serve as an intervention context if schools deliberately design tasks to provide manageable challenges, immediate accomplishment, and visible recognition. In this sense, the practical value of labor education lies not simply in offering labor experiences *per se*, but in helping students perceive these experiences as credible sources of support, competence, and meaning. Whether the same strategies would operate similarly in rural, county-level, central or western, or economically disadvantaged vocational schools remains to be tested in future research.

## 7 Limitations and future directions

This study has several limitations. First, the cross-sectional design precludes causal inferences. Although the hypothesized model was grounded in theory, the possibility of reverse causality, omitted variables, and endogeneity cannot be ruled out. For example, students with lower meaning in life or lower self-esteem may perceive family and school environments more negatively. This is particularly relevant because some components of family cumulative risk and labor education support perception were measured through students’ self-reports and may therefore be influenced by cognitive appraisal biases or negative self-schemas. Future research should employ longitudinal designs and lagged-variable models to control for prior levels of meaning in life and other psychological indicators. Cross-lagged panel models, random-intercept cross-lagged panel models, latent growth models, and multi-wave moderated mediation models would allow researchers to more rigorously examine the temporal ordering and reciprocal relations among family cumulative risk, self-esteem, labor education support perception, and meaning in life.

Second, all major variables were measured using self-report questionnaires at a single time point, which raises concerns about common method variance and social desirability bias. Although Harman’s single-factor test suggested that no single factor dominated the variance, this test is a relatively weak diagnostic technique and cannot definitively rule out common method bias. Therefore, some of the observed associations among family cumulative risk, self-esteem, labor education support perception, and meaning in life may have been inflated by shared measurement methods, response styles, or students’ general negative or positive appraisal tendencies. Future studies should adopt stronger procedural and statistical remedies, such as collecting data from multiple informants, including parent reports, teacher ratings, and peer reports; using objective or behavioral indicators where possible; temporally separating the measurement of predictors, mediators, moderators, and outcomes; including marker variables; and applying latent common method factor models. Longitudinal and multi-method designs would provide stronger evidence regarding the robustness of the observed associations.

Third, the sample was drawn exclusively from secondary vocational schools in Shenzhen city, one of China’s most economically developed cities, with relatively abundant educational funding, school-family resources, labor education support, and economic opportunities. Although a large proportion of the students in this study were non-local residents, most of them were migrant children who had moved to Shenzhen with their parents. Their family cumulative risk structure and access to labor education resources may differ substantially from those of secondary vocational school students in county-level, rural, central, or western regions. Therefore, the findings should not be directly generalized to all Chinese secondary vocational school students. Future research should expand the sampling scope and adopt stratified sampling across eastern, central, and western regions; different levels of urban and rural development; school types; and household registration groups across China. Multi-group analyses or multilevel modeling could be used to compare whether the path coefficients in the moderated mediation model differ across regions, school types, and student subgroups. Such efforts will substantially enhance the generalizability and external validity of the findings.

Fourth, the LESPS was newly developed for this study, and its psychometric evidence remains preliminary. Although the scale showed high internal consistency, acceptable confirmatory factor analytic fit, and preliminary discriminant validity in the present sample, it was developed and tested within a single Shenzhen-based sample. Therefore, the present findings cannot establish the scale’s generalizability, temporal stability, or full construct validity. Future research should validate the LESPS in independent samples from different regions, school types, and socioeconomic contexts. In particular, future studies should examine test–retest reliability, convergent validity with related constructs such as perceived school support, vocational identity support, and school belonging, as well as criterion-related and predictive validity with outcomes such as engagement in labor education, vocational identity, career adaptability, well-being, and meaning in life. Measurement invariance across gender, grade, household registration status, region, and school type should also be tested before meaningful group comparisons are made. In addition, future research could use item response theory or bifactor models to further examine item functioning and the dimensionality of the scale.

Fifth, the current model did not examine additional individual-level or school-level factors that may influence the observed associations. At the individual level, factors such as personality traits, resilience, vocational identity, career adaptability, academic self-concept, and mental health symptoms may affect how students perceive family risk, evaluate themselves, and interpret labor education support. At the school level, school climate, teacher-student relationships, peer support, counseling resources, school-enterprise cooperation, and the actual quality of labor education implementation may also be important. Moreover, although students were nested within schools, the present analyses were conducted at the individual level and did not explicitly account for school-level clustering or contextual variation. Future research should use multilevel designs to examine individual- and school-level influences simultaneously and to determine whether the proposed associations vary across schools, classes, and educational contexts.

## 8 Conclusion

This study examined the association between family cumulative risk and meaning in life among secondary vocational school students in Shenzhen, China, focusing on the statistically mediating role of self-esteem and the moderating role of labor education support perception in a theory-driven cross-sectional model. The results showed that family cumulative risk was negatively associated with meaning in life, and that the indirect association through self-esteem was significant. More importantly, two distinct moderation patterns were identified. In the association between family cumulative risk and self-esteem, labor education support perception appeared to function as a protective resource among students with relatively low family cumulative risk, consistent with the drop in the bucket model, but other explanations, including insufficient intervention intensity and contrast effects, are also plausible. Therefore, labor education support perception should be understood as a potentially meaningful but bounded school-based resource rather than a uniformly protective factor. In the association between self-esteem and meaning in life, labor education support perception appeared to compensate for lower self-esteem, consistent with the exclusion hypothesis. These findings highlight the importance of strengthening students’ internal psychological resources and optimizing perceived school-based support in secondary vocational education settings similar to Shenzhen, where relatively strong educational resources and labor education infrastructure are available. By emphasizing the identity-relevant function of labor education, this study suggests that the promotion of meaning in life among secondary vocational school students requires not only reducing family-related risks but also helping students transform vocational learning and labor participation into sources of competence, self-worth, and future purpose. Future longitudinal, multi-informant, multi-method, and multilevel research is needed to clarify the temporal, reciprocal, and contextual relations among family risk, self-esteem, labor education support perception, and meaning in life.

## Data Availability

The raw data supporting the conclusions of this article will be made available by the authors, without undue reservation.

## Footnotes

1 In China, “aesthetic education” is one of the five officially promoted dimensions of student development (moral, intellectual, physical, aesthetic, and labor education). It goes beyond art instruction to include the cultivation of aesthetic appreciation, creativity, and humanistic values.

## References

[B1] Bao Z. Li D. Zhang W. Wang Y. Sun W. Zhao L. (2014). Cumulative ecological risk and adolescents’ academic and social competence: The compensatory and moderating effects of sense of responsibility to parents. *Psychol. Dev. Educ.* 30 482–495.

[B2] Bao Z. Zhang W. Li D. Li D. Wang Y. (2013). School climate and academic achievement among adolescents: A moderated mediation model. *Psychol. Dev. Educ.* 29 61–70. 10.16187/j.cnki.issn1001-4918.2013.01.005

[B3] Baquero-Tomás M. Grau M. D. Moliner A. R. Sanchis-Sanchis A. (2023). Meaning in life as a protective factor against depression. *Front. Psychol.* 14:1180082. 10.3389/fpsyg.2023.1180082 37529311 PMC10389663

[B4] Baumeister R. F. (1991). *Meanings of Life.* New York, NY: Guilford Press.

[B5] Beck A. T. Haigh E. A. P. (2014). Advances in cognitive theory and therapy: The generic cognitive model. *Annu. Rev. Clin. Psychol.* 10 1–24. 10.1146/annurev-clinpsy-032813-153734 24387236

[B6] Benson P. L. Scales P. C. Hamilton S. F. Sesma A. (2007). “Positive youth development: Theory, research, and applications,” in *Handbook of Child Psychology*, eds Damon W. Lerner R. M. (Hoboken, NJ: Wiley), 894–941.

[B7] Bodner E. Bergman Y. S. Cohen-Fridel S. (2014). Do attachment styles affect the presence and search for meaning in life? *J. Happ. Stud.* 15 1041–1059. 10.1007/s10902-013-9462-7

[B8] Branje S. Hale W. W. Frijns T. Meeus W. (2010). Longitudinal associations between perceived parent-child relationship quality and depressive symptoms in adolescence. *J. Abnorm. Child Psychol.* 38 751–763. 10.1007/s10802-010-9401-6 20217211 PMC2902740

[B9] Bronfenbrenner U. Morris P. A. (1998). “The ecology of developmental processes,” in *Handbook of Child Psychology: Vol. 1. Theoretical Models of Human Development*, 5th Edn, ed. Lerner R. M. (New York, NY: Wiley), 993–1028.

[B10] Brook J. S. Brook D. W. De La Rosa M. Whiteman M. Montoya I. D. (1999). The role of parents in protecting Colombian adolescents from delinquency and marijuana use. *Arch. Pediatr. Adolesc. Med.* 153 457–464. 10.1001/archpedi.153.5.457 10323624

[B11] Buehler C. Gerard J. M. (2013). Cumulative family risk predicts increases in adjustment difficulties across early adolescence. *J. Youth Adolesc.* 42 905–920. 10.1007/s10964-012-9806-3 22915131

[B12] Chen Y. Zhang Y. Wang H. Wang K. (2023). The effect of campus exclusion on secondary vocational school students’ meaning in life: The serial mediating of psychological capital and negative coping style. *Psychol. Res.* 16 552–559. 10.19988/j.cnki.issn.2095-1159.2023.06.008

[B13] Cohen S. Wills T. A. (1985). Stress, social support, and the buffering hypothesis. *Psychol. Bull.* 98 310–357. 10.1037/0033-2909.98.2.310 3901065

[B14] Evans G. W. Li D. Whipple S. S. (2013). Cumulative risk and child development. *Psychol. Bull.* 139 1342–1396. 10.1037/a0031808 23566018

[B15] Fang J. Wen Z. Liang D. Li N. (2015). Moderation analysis based on multiple regression. *J. Psychol. Sci.* 38 715–720. 10.16719/j.cnki.1671-6981.2015.03.001

[B16] Fergus S. Zimmerman M. A. (2005). Adolescent resilience: A framework for understanding healthy development in the face of risk. *Annu. Rev. Public Health* 26 399–419. 10.1146/annurev.publhealth.26.021304.144357 15760295

[B17] Fritz J. de Graaff A. M. Caisley H. van Harmelen A.-L. Wilkinson P. (2018). A systematic review of amenable resilience factors that moderate and/or mediate the relationship between childhood adversity and mental health in young people. *Front. Psychiatry* 9:230. 10.3389/fpsyt.2018.00230 29971021 PMC6018532

[B18] Gao M. Teng Z. Wei Z. Jin K. Xiao J. Tang H. et al. (2022). Internet addiction among teenagers in a Chinese population: Prevalence, risk factors, and its relationship with obsessive-compulsive symptoms. *J. Psychiatr. Res.* 153 134–140. 10.1016/j.jpsychires.2022.07.003 35810603

[B19] Gard A. M. McLoyd V. C. Mitchell C. Hyde L. W. (2020). Evaluation of a longitudinal family stress model in a population-based cohort. *Soc. Dev.* 29 1155–1175. 10.1111/sode.12446 33953492 PMC8094580

[B20] Gibbons F. X. Buunk B. P. (1999). Individual differences in social comparison: Development of a scale of social comparison orientation. *J. Pers. Soc. Psychol.* 76 129–142. 10.1037/0022-3514.76.1.129 9972558

[B21] Gori A. Topino E. Svicher A. Di Fabio A. (2022). Towards meaning in life: A path analysis exploring the mediation of career adaptability in the associations of self-esteem with presence of meaning and search for meaning. *Int. J. Environ. Res. Public Health* 19:11901. 10.3390/ijerph191911901 36231203 PMC9565308

[B22] Halama P. Dedova M. (2007). Meaning in life and hope as predictors of positive mental health: Do they explain residual variance not predicted by personality traits? *Stud. Psychol.* 49 191–200.

[B23] Hayes A. F. (2017). *Introduction to Mediation, Moderation, and Conditional Process Analysis: A Regression-Based Approach.* New York, NY: Guilford Publications.

[B24] Hayes A. F. Montoya A. K. (2016). A tutorial on testing, visualizing, and probing an interaction involving a multicategorical variable in linear regression analysis. *Commun. Methods Meas.* 11 1–30. 10.1080/19312458.2016.1271116

[B25] Hobfoll S. E. (2011). Conservation of resource caravans and engaged settings. *J. Occup. Organ. Psychol.* 84 116–122. 10.1111/j.2044-8325.2010.02011.x

[B26] Hosokawa R. Katsura T. (2017). A longitudinal study of socioeconomic status, family processes, and child adjustment from preschool until early elementary school: The role of social competence. *Child Adolesc. Psychiatry Ment. Health* 11:62. 10.1186/s13034-017-0206-z 29270216 PMC5738164

[B27] Jiang H. (2020). “Three-Life” labor education: A practical exploration to promote the life growth of vocational school students. *Chin. Vocat. Tech. Educ.* 41 55–59. 10.3969/j.issn.1004-9290.2020.20.013

[B28] Jing Z. Ding F. (2024). Interaction between anxiety symptoms and decreased meaning in life: One possible pathway linking childhood trauma and depression—evidence from the network analysis. *J. Affect. Disord.* 355 440–449. 10.1016/j.jad.2024.03.108 38580034

[B29] Kaufman T. M. L. Kretschmer T. Huitsing G. Veenstra R. (2019). Caught in a vicious cycle? Explaining bidirectional spillover between parent-child relationships and peer victimization. *Dev. Psychopathol.* 32 11–20. 10.1017/S0954579418001360 30642413

[B30] Kaya A. Karataş N. Dalgiç A. I. (2023). The effect of individual values on self-esteem and meaning in life in adolescents: A cross-sectional study from Turkey. *Arch. Psychiatr. Nurs.* 46 8–13. 10.1016/j.apnu.2023.06.005 37813509

[B31] Kline P. (2015). *A Handbook of Test Construction (Psychology Revivals): Introduction to Psychometric Design.* London: Routledge.

[B32] Krauss S. Orth U. Robins R. W. (2020). Family environment and self-esteem development: A longitudinal study from age 10 to 16. *J. Pers. Soc. Psychol.* 119 457–478. 10.1037/pspp0000263 31535888 PMC7080605

[B33] Lakey B. Orehek E. (2011). Relational regulation theory: A new approach to explain the link between perceived social support and mental health. *Psychol. Rev.* 118 482–495. 10.1037/a0023477 21534704

[B34] Lambert N. M. Stillman T. F. Hicks J. A. Kamble S. Baumeister R. F. Fincham F. D. (2013). To belong is to matter: Sense of belonging enhances meaning in life. *Pers. Soc. Psychol. Bull.* 39 1418–1427. 10.1177/0146167213499186 23950557

[B35] Leng J. Cai H. Liu F. Shi X. Fan Z. (2025). Development and validation of the self-stigma scale for secondary vocational students (SSS-SVS). *Psychol. Res. Behav. Manag.* 18 91–104. 10.2147/PRBM.S500492 39830502 PMC11742590

[B36] Lerner R. M. (2004). Diversity in individual–context relations as the basis for positive development across the life span: A developmental systems perspective for theory, research, and application. *Res. Hum. Dev.* 1 327–346. 10.1207/s15427617rhd0104_5 42683494

[B37] Ma W. Gao P. Huang D. Zou W. (2022). Relationship between family cumulative risk factors and sense of life meaning of rural students: The mediating role of psychological capital. *Chin. J. Health Psychol.* 30 431–436. 10.13342/j.cnki.cjhp.2022.03.023

[B38] Masarik A. S. Conger R. D. (2017). Stress and child development: A review of the Family Stress Model. *Curr. Opin. Psychol.* 13 85–90. 10.1016/j.copsyc.2016.05.008 28813301

[B39] Miller I. W. Ryan C. E. Keitner G. I. Bishop D. S. Epstein N. B. (2000). The McMaster approach to families: Theory, assessment, treatment and research. *J. Fam. Ther.* 22 168–189. 10.1111/1467-6427.00145

[B40] Ministry of Education of the People’s Republic of China. (2020). *Guidelines for Labor Education in Primary, Secondary, and Higher Education Institutions (for Trial Implementation).* Available online at: https://www.gov.cn/zhengce/zhengceku/2020-07/15/content_5526949.htm (accessed July 7, 2020).

[B41] Ministry of Education of the People’s Republic of China. (2023). *Special Action Plan for Comprehensively Strengthening and Improving Student Mental Health Work in the New Era (2023-2025).* Available online at: https://www.gov.cn/zhengce/zhengceku/202305/content_6857361.htm (accessed April 20, 2023).

[B42] Ministry of Education’s Vocational Education Development Center. (2024). *China Vocational Education Development Report.* Beijing: Higher Education Press.

[B43] Moksnes U. K. Reidunsdatter R. J. (2019). Self-esteem and mental health in adolescents—level and stability during a school year. *Norsk Epidemiol.* 28 59–67. 10.5324/nje.v28i1-2.3052

[B44] National Bureau of Statistics of China. (2026). *Statistical Communiqué of the People’s Republic of China on the 2025 National Economic and Social Development.* Available online at: https://www.gov.cn/lianbo/202602/content_7059980.htm (accessed February 28, 2026).

[B45] Nell W. (2014). Exploring the relationship between religious fundamentalism, life satisfaction, and meaning in life. *J. Psychol. Afr.* 24 159–166. 10.1080/14330237.2014.903074

[B46] Orth U. (2018). The family environment in early childhood has a long-term effect on self-esteem: A longitudinal study from birth to age 27 years. *J. Pers. Soc. Psychol.* 114 637–655. 10.1037/pspp0000143 28182449

[B47] Orth U. Trzesniewski K. H. Robins R. W. (2010). Self-esteem development from young adulthood to old age: A cohort-sequential longitudinal study. *J. Pers. Soc. Psychol.* 98 645–658. 10.1037/a0018769 20307135

[B48] Oyserman D. Destin M. (2010). Identity-based motivation: Implications for intervention. *Couns. Psychol.* 38 1001–1043. 10.1177/0011000010374775 21516204 PMC3079278

[B49] Pérez-Fuentes M. C. Molero M. Gázquez J. Oropesa N. Simón M. Saracostti M. (2019). Parenting practices, life satisfaction, and the role of self-esteem in adolescents. *Int. J. Environ. Res. Public Health* 16:4045. 10.3390/ijerph16204045 31652560 PMC6844133

[B50] Podsakoff P. M. MacKenzie S. B. Lee J. Y. Podsakoff N. P. (2003). Common method biases in behavioral research: A critical review of the literature and recommended remedies. *J. Appl. Psychol.* 88 879–903. 10.1037/0021-9010.88.5.879 14516251

[B51] Qiu D. Ni J. Yang J. (2023). The influence of labor education participation on the subjective well-being of college students: Chain mediation effect of self-efficacy and healthy lifestyle. *Front. Psychol.* 14:1255030. 10.3389/fpsyg.2023.1255030 38078208 PMC10703146

[B52] Rosenberg M. (1965). *Society and the Adolescent Self-Image.* Princeton, NJ: Princeton University Press.

[B53] Rosenberg M. (2017). The self-concept: Social product and social force. *Soc. Psychol.* 30 593–624. 10.4324/9781315129723-19

[B54] Ryan R. M. Deci E. L. (2000). Self-determination theory and the facilitation of intrinsic motivation, social development, and well-being. *Am. Psychol.* 55 68–78. 10.1037/0003-066X.55.1.68 11392867

[B55] Shek D. T. L. (2005). A longitudinal study of perceived family functioning and adolescent adjustment in Chinese adolescents with economic disadvantage. *J. Fam. Issues* 26 518–543. 10.1177/0192513X04272618

[B56] Shenzhen Municipal Education Bureau. (2022). *Implementation Guidelines of the Shenzhen Municipal Education Bureau on Further Strengthening Labor Education in Primary, Secondary, and Higher Education Institutions.* Available online at: https://www.sz.gov.cn/cn/xxgk/zfxxgj/zcfg/content/post_9568535.html (accessed January 30, 2022).

[B57] Shonkoff J. P. Garner A. S. Siegel B. S. Dobbins M. I. Earls M. F. Garner A. S. et al. (2012). The lifelong effects of early childhood adversity and toxic stress. *Pediatrics* 129 e232–e246. 10.1542/peds.2011-2663 22201156

[B58] Steger M. F. Frazier P. Oishi S. Kaler M. (2006). The meaning in life questionnaire: Assessing the presence of and search for meaning in life. *J. Couns. Psychol.* 53 80–93. 10.1037/0022-0167.53.1.80

[B59] Tang M. (2023). The mediating role of meaning in life in the relationship between adverse childhood experiences and depressive symptoms in secondary vocational school students. *Appl. Educ. Psychol.* 4 70–75. 10.23977/appep.2023.041011

[B60] Wang H. Liu J. Li C. Fang A. Wang G. (2024). Research on the effect mechanism of career-specific parental support promoting meaning in life of Chinese higher vocational college students. *Behav. Sci.* 14:1172. 10.3390/bs14121172 39767313 PMC11673999

[B61] Wang W. L. Zhou Y. Q. Chai N. N. Li G. H. Liu D. W. (2020). Mediation and moderation analyses: Exploring the complex pathways between hope and quality of life among patients with schizophrenia. *BMC Psychiatry* 20:22. 10.1186/s12888-020-2436-5 31941476 PMC6964047

[B62] Wu S. Duan J. Luo M. (2024). Evaluating and analyzing student labor literacy in China’s higher vocational education: An assessment model approach. *Front. Educ.* 9:1361224. 10.3389/feduc.2024.1361224

[B63] Xiang Y. Li L. Yang Q. Fang Y. Xu W. Ding X. et al. (2024). Career adaptability and correlating factors among secondary vocational nursing students in China. *Front. Public Health* 12:1377323. 10.3389/fpubh.2024.1377323 39360255 PMC11445154

[B64] Xiong J. Fang X. Wang J. Xie W. Liu M. Niu G. (2024). Family cumulative risk, life satisfaction, and anxiety and depression in adolescents: A developmental cascades model. *J. Adolesc.* 96 1445–1457. 10.1002/jad.12283 38783637

[B65] Xiong J. Hai M. Huang F. Xin L. Xu Y. (2020). Family cumulative risk and mental health in Chinese adolescents: The compensatory and moderating effects of psychological capital. *Psychol. Dev. Educ.* 36 94–102. 10.16187/j.cnki.issn1001-4918.2020.01.11

[B66] Xu M. Niu T. (2024). Effects of labor participation on subjective well-being of primary school students: The analysis of the chain mediating effects of family support and sense of meaning in life. *Educ. Meas. Eval.* 2 38–48.

[B67] Xue E. Li J. (2022). Exploring the type-based vocational education system: insights from China. *Educ. Philos. Theory* 54 1670–1680. 10.1080/00131857.2021.1934668

[B68] Xuede X. Xinyu Z. Ye L. Zihan L. Yuewang Z. Yifan W. et al. (2023). Analysis of the role of labor education in the growth education of college students. *Front. Educ. Res.* 6:50–55. 10.25236/FER.2023.062008

[B69] Yu X. Lin X. Lu S. Zhang H. Bu A. (2025). Stability and transformation: The impact of family socioeconomic status and club cadre experience on the sense of meaning in life of secondary vocational school students. *Educ. Sci. Forum* 41 69–75. 10.3969/j.issn.1673-4289.2025.30.014

[B70] Yun A. Cai Y. Hu Y. (2025). The relationship between Chinese vocational college students’ adverse childhood experience and meaning in life: The mediating role of self-esteem and the moderating role of humorous coping. *Front. Psychol.* 16:1467780. 10.3389/fpsyg.2025.1467780 40309205 PMC12040953

[B71] Zeng J. Zhang M. Li Y. Chen Y. (2026). Longitudinal associations between family socioeconomic status and adolescent depressive symptom trajectories in China: A chain multiple mediation model. *J. Youth Adolesc.* 55 1804–1818. 10.1007/s10964-026-02355-4 41945248 PMC13328128

[B72] Zhao J. Zhao H. Zhou A. (2023). Negative parenting styles and psychological crisis in adolescents: Testing a moderated mediating model of school connectedness and self-esteem. *Behav. Sci.* 13:929. 10.3390/bs13110929 37998676 PMC10669031

[B73] Zhou H. Long L. (2004). Statistical remedies for common method biases. *Adv. Psychol. Sci.* 12 942–950. 10.3969/j.issn.1671-3710.2004.06.018

[B74] Zimmerman M. A. Stoddard S. A. Eisman A. B. Caldwell C. H. Aiyer S. M. Miller A. (2013). Adolescent resilience: Promotive factors that inform prevention. *Child Dev. Perspect.* 7 215–220. 10.1111/cdep.12042 24288578 PMC3839856

