# Supplementary material for: Family cumulative risk and meaning in life among Chinese secondary vocational school students: the mediating role of self-esteem and the moderating role of labor education support perception
Source: Front Psychol. 2026 Sep 8;17:1882107. doi: 10.3389/fpsyg.2026.1882107 (PMC13593749; doi:10.3389/fpsyg.2026.1882107)
Supplement: Supplementary file 1 [file Supplementary_file_1.docx]

Supplementary Material

1 **Supplementary material for family cumulative risk measurement**

## 1.1 **Family structure risk**

① Family type. Following the study by Dong and Lin (2011), the family type was measured using a single item: "Who currently lives with you?" Those not living with their biological parents were considered at risk and coded as 1; others were coded as 0.

② Parent-child separation. Following Xiong et al. (2020), two items were used: "In the past 6 months, has your father/mother been working away from home for extended periods and unable to reunite with you?" If either parent was working away from home, the individual was considered at risk and coded as 1; otherwise, they were coded as 0.

③ Household registration status. One item was used: "Where is your household registration (hukou) located?" Those with a hukou in “Shenzhen” were considered at low risk and coded as 0; all others were considered at risk.

## 1.2 Family resource deficiency risk

① Parental educational attainment. Following Bao et al. (2014), a two-item measurement was used. If both parents' educational attainment was below "high school (including vocational high school and technical school)," it was considered risky and coded as 1; otherwise, it was coded as 0.

② Parental employment. Following Dong and Lin (2011), a two-item measurement was used: "Does your father/mother have stable employment?" If either parent’s situation was "no," it was considered risky and coded as 1; otherwise, it was coded as 0.

③ Parental health. Two items were used: "What is your parents' health status?" Options included "very poor," "poor," "average," "good," and "very good." If either parent's health status was "very poor" or "poor," it was coded as 1 (risky); otherwise, it was 0 (no risk).

④ Family economic status. The Economic Strain Scale, developed by Wadsworth and Compas (2002) and revised by Wang et al. (2010), was used. It consists of four items scored on a 5-point Likert scale. The higher the score, the more difficult the household's economic situation. Scores at or above the 75th percentile were coded as 1; all others were coded as 0.

## 1.3 Family atmosphere risk

① Parental interaction and involvement. Following the research of Evans et al. (2013), a single item was used to measure "On average, how much time do you spend each week engaging in interactive activities (playing, exercising, talking) with your parents?" Participants scoring below or equal to the 25th percentile were considered at risk and coded as 1; the rest were coded as 0.

② Family cohesion. The cohesion dimension of the Family Adaptability and Cohesion Evaluation Scale-II (Olson et al., 1982) was used for measurement. It consists of 16 items scored on a 5-point scale. The Cronbach’s α coefficient for this scale was 0.90, indicating good reliability. Scores below or equal to the 25th percentile were considered at risk and coded as 1; the rest were coded as 0.

③ Parent-child relationship. The Closeness to Parents Scale, developed by Buchanan et al. (1991), consisted of 18 items scored on a 5-point scale, measuring the quality of parent-child relationships between vocational school students and their fathers and mothers, respectively. Higher scores indicated a closer relationship between the adolescent and their parents. The scale's Cronbach's α coefficients were 0.93 (father-child subscale) and 0.94 (mother-child subscale), indicating good reliability. Scores at or below the 25th percentile were coded as 1 (at risk); all others were coded as 0 (not at risk).

# 2 References

Bao, Z., Li, D., Zhang, W., Wang Y., Sun W., and Zhao, L. (2014). Cumulative ecological risk and adolescents' academic and social competence: the compensatory and moderating effects of sense of responsibility to parents. Psychol. Dev. Educ. 30, 482-495.

Buchanan, C. M., Maccoby, E. E., and Dornbusch, S. M. (1991). Caught between parents: adolescents' experience in divorced homes. Child Dev. 62, 1008-1029. doi: 10.2307/1131149

Dong, Q., and Lin, C. (2011). Introduction to Standardized Tests of Psychological Development for Chinese Children and Adolescents. Beijing: Science Press.

Evans, G. W., Li, D., and Whipple, S. S. (2013). Cumulative risk and child development. Psychol. Bull. 139, 1342-1396. doi: 10.1037/a0031808

Olson, D., McCubbin, H., Barnes, H., Larsen, A., Muxen, M., and Wilson, M. (1982). Families Inventories: Inventories Used in a National Survey of Families Across the Family Life-Cycle. St. Paul, MN: Family Social Science, University of Minnesota.

Wadsworth, M. E., and Compas, B. E. (2002). Coping with family conflict and economic strain: the adolescent perspective. J. Res. Adolesc. 12, 243-274. doi: 10.1111/1532-7795.00033

Wang, J., Li, D., and Zhang, W. (2010). Adolescence’s family financial difficulty and social adaptation: coping efficacy of compensatory, mediation, and moderation effects. J. Beijing Norm. Univ. (Soc. Sci.) 4, 22-32. doi: 10.3969/j.issn.1002-0209.2010.04.002

Xiong, J., Hai, M., Huang, F., Xin, L., and Xu, Y. (2020). Family cumulative risk and mental health in Chinese adolescents: the compensatory and moderating effects of psychological capital. Psychol. Dev. Educ. 36, 94-102. doi: 10.16187/j.cnki.issn1001-4918.2020.01.11
